# Predictive Factors of Conception and the Cumulative Pregnancy Rate in Subfertile Couples Undergoing Timed Intercourse With Ultrasound

**DOI:** 10.3389/fendo.2021.650883

**Published:** 2021-04-15

**Authors:** So Hyun Ahn, Inha Lee, SiHyun Cho, Hye In Kim, Hye Won Baek, Jae Hoon Lee, Yun Jeong Park, Heeyon Kim, Bo Hyon Yun, Seok Kyo Seo, Joo Hyun Park, Young Sik Choi, Byung Seok Lee

**Affiliations:** ^1^ Department of Obstetrics and Gynecology, Severance Hospital, Yonsei University College of Medicine, Seoul, South Korea; ^2^ Institute of Women’s Life Medical Science, Yonsei University College of Medicine, Seoul, South Korea; ^3^ Department of Obstetrics and Gynecology, Gangnam Severance Hospital, Yonsei University College of Medicine, Seoul, South Korea; ^4^ Department of Obstetrics and Gynecology, Yongin Severance Hospital, Yonsei University College of Medicine, Seoul, South Korea

**Keywords:** timed intercourse, ultrasonography, predictive factor, cumulative pregnancy rate, subfertile couples

## Abstract

The aim of this study was to determine predictive factors for pregnancy and assess the cumulative pregnancy rate (CPR) and live birth rate (CLBR) in subfertile couples undergoing timed intercourse (TI) using ultrasound. This retrospective cohort study included 285 women (854 cycles) who started TI with ultrasound between January 2017 and October 2019. The overall clinical pregnancy rate was 28.1% (80/285) per couple and 9.4% (80/854) per cycle. Pregnant women had a higher body mass index (BMI), higher percentage of irregular menstrual cycles, a shorter duration of subfertility, lower serum follicle-stimulating hormone levels, and higher anti-Müllerian hormone levels than non-pregnant women. A longer duration of subfertility (≥24 months *vs.* <12 months; odds ratio: 0.193; 95% confidence interval: 0.043-0.859) and endometriosis (*vs.* ovulatory factors; odds ratio: 0.282; 95% confidence interval: 0.106-0.746) as causes of subfertility were unfavorable factors that independently affected clinical pregnancy. In subgroup analysis, old age ≥ 35 years [*vs.* < 35 years; odds ratio: 0.279; 95% confidence interval: 0.083-0.938), a longer duration of infertility ≥24 months (*vs.* <24 months; odds ratio: 0.182; 95% confidence interval: 0.036-0.913) and a higher BMI ≥ 25 kg/m^2^(*vs.* >25 kg/m^2^; odds ratio: 3.202; 95% confidence interval: 1.020-10.046) in couples with ovulatory factor and a longer duration of infertility ≥24 months (*vs.* <24 months; odds ratio: 0.185; 95% confidence interval: 0.042-0.819) in couples with non-ovulatory factors were significant independent predictive factors for pregnancy. No significant differences were found in the cycle characteristics between pregnant and non-pregnant women. The CPR substantially increased during the first three cycles and significantly increased until the sixth cycle. No significant increase was observed in the CPR after the sixth cycle. The CLBRs substantially increased during the first three cycles and significantly increased until the fourth cycle. No significant increase was observed in the CLBRs after the fifth cycle. When comparing CPRs and CLBRs according to subfertile causes, CRPs was significantly different and CLBRs was different with borderline significance. Our findings may indicate that women with a longer duration of subfertility or subfertility due to endometriosis have poor outcomes during TI with ultrasound. Women who failed to achieve conception by the fourth or fifth cycle of TI with ultrasound may be encouraged to consider advancing to the next treatment strategy.

## Introduction

Approximately 85-90% of healthy young couples conceive within 1 year, most within 6 months (1, 2). Infertility therefore affects approximately 10-15% of couples and represents a significant part of clinical practice (3). A normal sperm can maintain the ability to fertilize an egg for at least 3 and up to 5 days, but an oocyte can be fertilized for about 12-24 hours after ovulation. Consequently, virtually all pregnancies result from sexual intercourse occurring sometime within the 6-day interval, the fertile window, ending on the day of ovulation (4, 5). Therefore, timed intercourse (TI) during this fertile window, is one of the simple and commonly prescribed treatments for couples who want to become pregnant.

However, even when intercourse is carefully timed, cycle fecundity does not exceed approximately 35% in normally fertile couples (4, 6). While continuing to attempt pregnancy with TI, women should also consider age-related decline in fertility over time (7). The anxiety of repeated unsuccessful conceptions and pressures of timed coitus are stresses for both partners that might also reduce the chances of pregnancy (8, 9). Therefore, attempts using TI for a reasonable sustainable period is important to avoid both over- and under-management for couples who wish to become pregnant.

Previous studies on TI predicted ovulation by indirect indexes such as calendar charting, tracking basal body temperature, cervical secretion investigation, and urinary hormone measurement, including the levels of luteinizing hormone (LH) or estrogen.

Fertility awareness-based methods (FABMs) estimate the fertile time by observing fertility signs such as cervical secretions and basal body temperature or monitoring cycle length (10). Those parameters have long been used because of simplicity and non-invasiveness. However, they are difficult to interpret and need an educational component for proper use (11, 12). A randomized study comparing cervical mucus monitoring versus frequent intercourse found no benefit to the pregnancy rate in the mucus monitoring group (13). Mobile fertility tracking applications predict fertile days based on one or more parameters of the FABMs. However, most applications were not developed or sponsored by health care professionals and assumed fertile windows regardless of average cycle length or between-cycle variability. Therefore, there are concerns about reliability and effectiveness (14, 15).

Urinary ovulation predictor kits that monitor LH and or estrone-3-glucuronide (E1G) in urine are convenient, non-invasive methods to detect ovulation. However, they do not allow the prospective determination of the entire fertile window (16, 17) and may present a relatively high number of false negatives when peak LH concentrations are low or when the LH surge duration is too short to be detected (18, 19). False-positive test results also occur in approximately 7% of cycles (20). In a systemic review, three randomized controlled trials showed that urinary ovulation kits might increase pregnancy rates compared with not using them (pooled RR: 1.36; 95% CI: 1.07-1.73) (21). However, the quality of evidence was low to very low with a very small number of total participants. New tests that combine existing indirect indices using software applications or detect new materials are being studied to increase the predictive value (22–24); clinical usefulness is expected in the future.

The most direct method, laparoscopy, is technically difficult to perform routinely. Another direct method is to detect the maximum growth of dominant follicles close to ovulation and its subsequent reduction in size by high-resolution transvaginal ultrasonography (TVUS) (25). The time of ovulation can be determined precisely through follicle monitoring using ultrasound (26–29). Ultrasonography is recognized as a reference test for ovulation detection and is used primarily in assisted reproductive technology (ART) that requires a clear fertility window (30). Although information on predictive factors of conception, the cumulative pregnancy rate (CPR) and the cumulative live birth rate (CLBR) in couples undergoing TI with ultrasound may help decide when to advance to the next treatment, few data are available. Our study aimed to determine predictive factors for pregnancy and assess the CPR and the CLBR in couples undergoing TI with ultrasound.

## Materials and Methods

The medical records were retrieved from 285 couples who started TI with ultrasound at the Severance Hospital Infertility Clinics from January 2017 to October 2019. The inclusion criteria were couples trying to conceive, and unable to conceive for more than 6 months of usual sexual intercourse. Women aged between 27 and 43 years were included. Only women with at least one patent tube were included. Couples who currently received subfertility treatments or those with severe male factor infertility including azoospermia or severe oligoasthenoteratozoospermia (<10 million total motile sperm count or <2% strict normal morphology) were excluded. Women without a heterosexual partnership were excluded. Among all started cycles, the cycles in which intercourse was missed and ovulation did not occur were not included for the analysis. The duration of subfertility was defined as the interval in months from the discontinuation of contraceptive activities until registration at the fertility center. The menstrual cycle was considered regular if the cycle period was between 24 and 38 days, with interval variation less than 20 days. Our study was approved by the Institutional Review Board of Severance Hospital, Yonsei University College of Medicine (No. 2020-0312).

Patients visited the clinic on the day 2 to 5 of the menstrual cycle and had undergone medical and reproductive history recording, serum hormone assays including follicle-stimulating hormone (FSH), LH, estradiol (E_2_), anti-Müllerian hormone (AMH), thyroid-stimulating hormone, prolactin, and baseline TVUS. Hysterosalpingography was performed 2-5 days after the end of menstruation, and analysis of the partner’s semen was performed after 2-4 days of abstinence. Fertility is mostly affected by ovulatory factors, uterine factors, endometriosis, male factors, and unexplained subfertility. Ovulatory factors include irregular menstrual cycles or anovulation. Uterine factors include a uterine myoma larger than 6 cm or with distortion of the uterine cavity, adenomyosis or endometrial polyp. Endometriosis was diagnosed by ultrasound or laparoscopy. Male factors include at least two semen analyses 4 weeks apart showing a sperm count <15×10^6^/ml or <40% motility or <4% strict normal morphology. If no abnormal findings were found, they were classified as unexplained subfertility.

Each cycle was individualized at the discretion of the physician according to standard institutional protocols. In couples with ovulatory factor, ovulation induction was initiated from the first cycle. In couples with non-ovulatory causes, timed intercourse was performed in natural cycle or ovulation induction cycle. Ovulation induction was initiated with clomiphene citrate (CC) or letrozole by the preference of physician and couples. The gonadotropin was added if the follicular growth was inadequate in the present or previous cycle by the physician’s preference. Follicular growth was monitored by TVUS from days 11-15 of the menstrual cycle until the follicle reached 18-20 mm. When the diameter of at least one follicle reached 18 mm, 5,000 IU of urinary human chorionic gonadotropin (hCG) (IVF-C; LG Life Sciences, Republic of Korea) or 250 μg of recombinant hCG (Ovidrel liquid; Merck, Switzerland) was administered intramuscularly or subcutaneously. The patients were advised to have intercourse 34-40 hours after hCG administration. If the dominant follicle was confirmed but did not reach 18-20mm and further follow-up was not possible, patients were advised to have intercourses for three consecutive days when the follicle diameter is expected to reach 20-24 mm. For the ovulation induction cycles, CC (Clomiphene 50-150 mg/d; Young Poong Pharma, Seoul, Republic of Korea) or letrozole (Femara 5 mg/day; Novartis, Basel, Switzerland) was administered for 5 days starting from menstrual cycle day 3 to 5. If necessary, 75 or 150 IU of recombinant FSH (Follitrope, LG Life Sciences) or human menopausal gonadotropin (IVF-M; LG Life Sciences) was also administered.

As soon as the patients missed the next period, they performed a self-administered urine pregnancy test. Clinical pregnancy was defined as the presence of one or more gestational sacs by transvaginal ultrasound at 3 to 4 weeks after ovulation. Miscarriage was defined as fetal demise or the absence of a fetal heart rate before 20 weeks. If pregnancy was not achieved, the next cycle was started.

To compare the clinical characteristics and parameters between the pregnant and non-pregnant groups or cycles, the two-sample t-test was used for continuous variables and chi-squared or Fisher’s exact test was used for categorical variables as appropriate. Logistic regression analysis was performed to determine significant variables that could independently contribute to pregnancy. A value of *p*<0.05 was considered statistically significant. The CPRs and CLBRs were analyzed using McNemar’s test, and the significance level was corrected using the Bonferroni method. The CPRs and CLBRs from groups with different causes of subfertility were compared using generalized estimating equation (GEE), and a value of *p*<0.05 was considered statistically significant. All statistical analyses were conducted using IBM SPSS ver. 25.0 (IBM corp., Armonk, NY, USA), SAS version 9.4 (SAS Institute, Cary, NC, USA) and R package version 4.0.2 (http://www.R-project.org).

## Results

The overall clinical pregnancy rate was 28.1% (80/285) per couple and 9.4% (80/854) per cycle. The overall live birth rate was 23.2% (66/285) per couple and 7.7% (66/854) per cycle. Ten pregnancies resulted in miscarriages during the first trimester, 3 were ectopic (2.5%), and 1 was a stillbirth (1.25%). Of the 66 live births, 5 were twin pregnancies (7.6%).

The clinical characteristics of the pregnant and non-pregnant groups are shown in **Table 1**. The parity, number of cycles, basal serum LH, E_2_, tubal patency, and semen parameters were comparable between the groups. The pregnant group had a younger age, higher body mass index (BMI), higher percentage of irregular menstrual cycles, a shorter duration of subfertility, lower serum FSH levels, and higher AMH levels than the non-pregnant group. The causes of subfertility were significantly different between the pregnant and non-pregnant groups.

**Table 1:** Comparison of the clinical characteristics between the pregnant and non-pregnant groups.

Variables	Total (n=285)		Pregnant group (n=80)	Non-pregnant group (n=205)	*p*-value
Age of women (years)	33.68 ± 3.69		32.88 ± 3.32	33.99 ± 3.79	0.045
Body mass index (kg/m^2^)	21.94 ± 3.67		22.57 ± 3.25	21.69 ± 3.82	0.008
Menstrual cycle					0.006
Regular	158 (55.4%)		34 (42.5%)	124 (60.5%)	
Irregular	127 (44.6%)		46 (57.5%)	81 (39.5%)	
Previous pregnancy					0.557
No	203 (71.2%)		59 (73.8%)	144 (70.2%)	
Yes	82 (28.8%)		21 (26.3%)	61 (29.8%)	
Duration of subfertility (months)	15.39 ± 19.36		11.33 ± 11.1	17.16 ± 21.58	0.003
Causes of subfertility					0.018
Ovulatory	103 (36.1%)		41 (51.25%)	62 (30.2%)	
Unexplained	79 (27.7%)		18 (22.5%)	61 (29.8%)	
Uterine	59 (17.54%)		14 (17.5%)	45 (22.0%)	
Endometriosis	36 (12.6%)		6 (7.5%)	30 (14.6%)	
Mild male factor	8 (2.8%)		1 (1.25%)	7 (3.4%)	
Mean number of cycles	3.03 ± 2.33		2.8 ± 2.479	3.03 ± 2.267	0.47
Basal serum FSH (mIU/mL)	8.61 ± 7.94		7.14 ± 3.32	9.04 ± 8.71	0.008
Basal serum LH (mIU/mL)	7.34 ± 5.15		7.96 ± 4.76	7.10 ± 5.28	0.598
Basal serum E_2_ (pg/mL)	60.61 ± 62.92		62.11 ± 44	60.03 ± 68.91	0.915
AMH (ng/dL)	5.58 ± 5.33		7.11 ± 6.22	4.99 ± 4.67	0.008
Age of husband (years)	36.05 ± 4.86		35.75 ± 4.49	36.20 ± 5.05	0.381
Semen analysis					
TMSC	105.39 ± 108.15		107.17 ± 127.73	105.07 ± 104.77	0.254
Motility (%)	51.10 ± 25.19		52.61 ± 22.53	50.83 ± 25.72	0.974
Strict morphology (%)	7.03 ± 14.46		6.42 ± 12.95	7.15 ± 14.77	0.649
Concentration (×10^6^/ml)	58.10 ± 47.50		47.66 ± 34.85	60.05 ± 49.38	0.209

Data are presented as the mean ± S.D., or number of cases (%).

FSH, follicle-stimulating hormone; LH, luteinizing hormone; E_2_, estradiol; AMH, anti-Müllerian hormone; TMSC, total motile sperm count.

Logistic regression analysis for predicting pregnancy is shown in **Table 2**. In univariate analysis, female age ≥35 years, the duration of subfertility, serum basal FSH levels and a longer duration of subfertility (≥24 months) as well as unexplained infertility, uterine factors, and endometriosis as a cause of subfertility showed a lower probability to achieve conception. In multivariate analysis, a longer duration of subfertility [≥24 months *vs.* <12 months; odds ratio (OR): 0.19; 95% confidence interval (CI): 0.043-0.846; *p*=0.029] and endometriosis as a cause of subfertility (*vs.* ovulatory factors; OR: 0.291; 95% CI: 0.110-0.774; *p*=0.013) remained as independent unfavorable predictors for clinical pregnancy. The participants were also stratified into two groups according to the cause of subfertility (ovulatory factor and non-ovulatory factor). Logistic regression analysis for predicting pregnancy was conducted according to ovulatory factor and non-ovulatory factor (**Table 3**). In univariate analysis, a lower BMI (<25 kg/m^2^) and a longer duration of subfertility (≥24 months) showed a lower probability to achieve conception of subfertile couples with ovulatory factor. In multivariate analysis, female age ≥ 35 years (*vs.* ≥35 years; OR: 0.29; 95% CI: 0.086-0.947; *p*=0.040) and duration of subfertility ≥ 24 months (*vs.* <24 months; OR: 0.193; 95% CI: 0.041-0.908; *p*=0.037) remained as independent unfavorable factors for clinical pregnancy. Higher BMI ≥25 kg/m^2^ remained as independent favorable factor (*vs.* <25 kg/m^2^; OR: 3.202; 95% CI: 1.020-10.046; *p*=0.046). For subfertile couples with non-ovulatory factors, a longer duration of subfertility ≥24 months was an independent unfavorable predictor of clinical pregnancy (*vs.* <24 months; odds ratio (OR): 0.2; 95% confidence interval(CI): 0.042-0.819; *p*=0.042) (**Table 3**).

**Table 2:** Logistic regression analysis of the clinical characteristics to predict pregnancy in women undergoing timed coitus.

Variables			Univariate	*p*-value	Multivariate		*p*-value
			OR (95% CI)		OR (95% CI)		
Age of women (years)							0.276
	<35		1		1		
	≥35		0.558 (0.320-0.976)	0.041	0.715 (0.39-1.309)		
Duration of subfertility (months)							0.079
	<12		1		1		
	12-24		0.852 (0.451-1.61)	0.621	0.766 (0.399-1.472)		0.424
	≥24		0.196 (0.045-0.859)	0.031	0.19 (0.043-0.846)		0.029
Causes of subfertility							0.062
	Ovulatory		1		1		
	Unexplained		0.446 (0.231-0.861)	0.016	0.524 (0.265-1.036)		0.063
	Uterine		0.470 (0.229-0.965)	0.040	0.529 (0.254-1.101)		0.089
	Endometriosis		0.302 (0.116-0.791)	0.015	0.291 (0.110-0.774)		0.013
	Male factor		0.216 (0.026-1.822)	0.159	0.307 (0.034-2.747)		0.291
Body mass index (kg/m^2^)							
	<25		1				
	≥25		1.775 (0.889-3.545)	0.104			
Serum basal FSH (mIU/mL)							0.101
	<10		1		1		
	>10		0.295 (0.101-0.866)	0.026	0.394 (0.13-1.2)		

FSH, follicle-stimulating hormone.

**Table 3:** Logistic regression analysis of the clinical characteristics to predict pregnancy in timed intercourse of subfertile couples with ovulatory factor and non-ovulatory factor.

Variables	Ovulatory factor				Non-ovulatory factor				
	Univariate		Multivariate		Univariate		Multivariate		
	OR(95% CI)	*p*-value	OR(95% CI)	*p*-value	OR(95% CI)	*p*-value	OR(95% CI)		*p*-value
Age of women (years)									
<35	1	0.051	1	0.039	1	0.266			
≥35	0.340 (0.115-1.004)		0.279 (0.083-0.938)		0.583 (0.225-1.509)				
Duration of subfertility (months)									
<24	1	0.037	1	0.038	1	0.02	1		0.042
≥24	0.193 (0.041-0.908)		0.182 (0.036-0.913)		0.173 (0.040-0.757)		0.185 (0.042-0.819)		
BMI (kg/m^2^)									
<25	1	0.046	1	0.046	1	0.971			
≥25	2.918 (1.018-8.366)		3.202 (1.020-10.046)		0.980 (0.339-2.834)				
Serum basal FSH (mIU/mL)									
<10	1	0.817							
≥10	0.750 (0.066-8.550)								
Causes									
Unexplained					1				
Uterine factor					1.054	0.897			
					(0.475-2.341)				
Endometriosis					0.678	0.456			
					(0.244-1.884)				
Male factor					0.484	0.513			
					(0.056-1.884)				
Serum AMH (ng/mL)									
<1.5					1	0.067	1		0.086
≥1.5					2.589 (0.936-7.163)		2.469 (0.880-6.927)		

BMI, Body mass index; FSH, follicle-stimulating hormone; AMH, anti-Müllerian hormone.

No significant differences were observed in the cycle characteristics, including ovulation induction regimen, endometrial thickness, and pattern, and the number of follicles with a diameter ≥14 mm, between the pregnant and non-pregnant cycles (**Table 4**).

**Table 4:** Comparison of the cycle characteristics between the pregnant and non-pregnant cycles.

Variables	Total	Pregnant cycles	Non-pregnant cycles	*p*-value
Cycle Number	854	80 (9.4%)	774 (90.6%)	
Regimen				0.629
Natural cycle	131 (15.3%)	10 (12.5%)	121 (15.6%)	
CC	281 (32.9%)	27 (33.75%)	254 (32.8%)	
Letrozole	372 (43.6%)	36 (45%)	336 (43.4%)	
CC + gonadotropin	28 (3.3%)	1 (1.25%)	27 (3.5%)	
Letrozole + gonadotropin	42 (4.9%)	6 (7.5%)	36 (4.7%)	
hCG injection				0.133
No	554 (64.9%)	58 (72.5%)	496 (64.1%)	
Yes	300 (35.1%)	22 (27.5%)	278 (35.9%)	
EM pattern				0.252
non-triple	99 (11.7%)	6 (7.7%)	93 (12.1%)	
triple	750 (88.3%)	72 (92.3%)	678 (87.9%)	
EM polyp				0.240
yes	132 (15.5%)	16 (20%)	116 (15%)	
no	721 (84.5%)	64 (80%)	657 (85%)	
EM thickness (mm)				0.37
<7 mm	103 (12.1%)	7 (9%)	96 (12.5%)	
≥7 mm	746 (87.9%)	71 (91%)	675 (87.5%)	
Number of follicles				
≥14 mm	1.60 ± 1.08	1.65 ± 0.978	1.6 ± 1.087	0.447

Data are presented as the mean ± S.D., or number of cases (%).

CC, clomiphene citrate; hCG, human chorionic gonadotropin; EM, endometrium.

**Figure 1:**
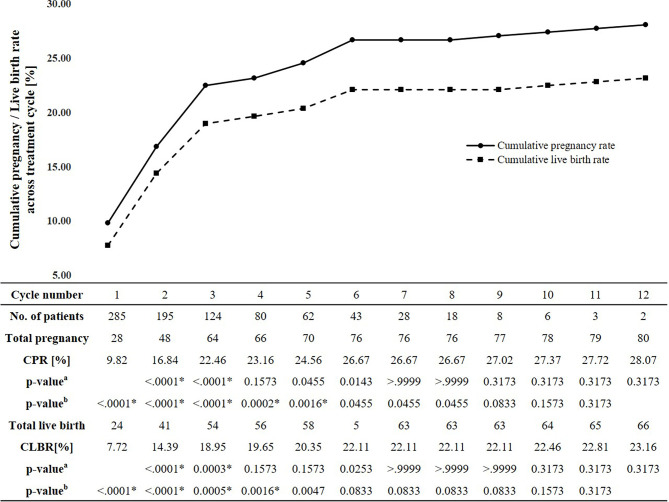
Estimated cumulative pregnancy and live birth rates per cycle and trend. CPR, Cumulative pregnancy rate; CLBR, Cumulative live birth rate. ^a^McNemar’s test: comparing the cumulative pregnancy rate in each cycle with the cumulative pregnancy rate in the previous cycle. ^b^McNemar’s test: comparing the cumulative total cumulative pregnancy rates and pregnancy rate in each cycle. *The significance level corrected by Bonferroni method is 0.05/11 = 0.0045.

**Figure 2:**
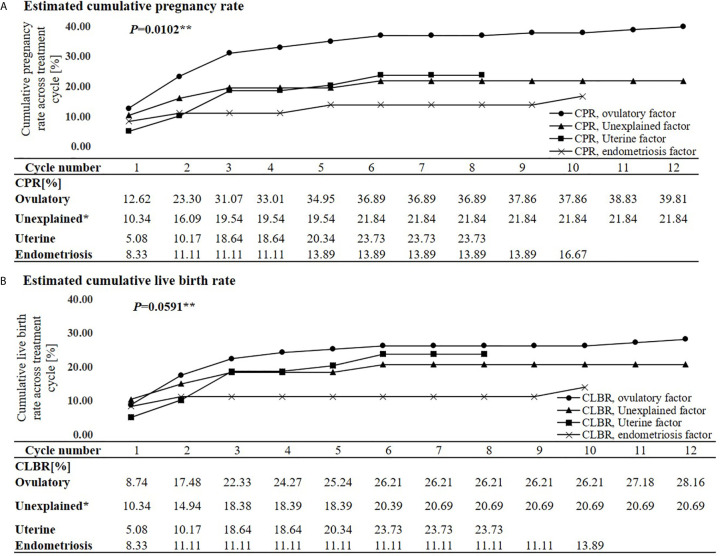
Estimated cumulative pregnancy rate **(A)** and live birth rate **(B)** per each cycle and trends according to subfertile causes. CPR, Cumulative pregnancy rate; CLBR, Cumulative live birth rate. *Eight cases of the mild male factor data were combined with the unexplained factor group. **Generalized estimating equation.

The estimated CPRs at 1, 3, 6, 9, and 12 cycle(s) were 9.8%, 22.5%, 26.7%, 27.0%, and 28.1%, respectively (**Figure 1**). Comparing the difference in the CPR from the previous cycle in each cycle, the CPR substantially increased during the first three cycles and significantly increased until the fifth cycle. No significant increase was found in the CPR after the sixth cycle. In the sixth cycle, the maximal CPR was approached, and no significant difference was observed between the maximal CPR and CPR of the additional cycles. The estimated CLBRs at 1,3,6, and 12 cycle(s) were 7.7%, 19.0%, 22.1%, 23.2%, respectively (**Figure 1**). The CLBRs also substantially increased during the first three cycles. There was a significant increase until the fourth cycle but no significant difference after the fifth cycle. The estimated CPRs (**Figure 2A**) and CLBRs (**Figure 2B**) in each group of ovulatory factor, uterine factor, endometriosis factor and unexplained factor which is combined with mild male factor showed similar trends to overall results. The CPRs and CLBRs of the ovulatory factor were the highest, and those of endometriosis was the lowest. The CPRs were statistically different (*p* = 0.0102) and CLBRs were different with borderline significance among these groups (*p*=0.0591).

## Discussion

In this single-center retrospective cohort study, 854 cycles in 285 patients were analyzed to determine predictive factors for pregnancy and evaluate the CPR and the CLBR in couples undergoing TI with ultrasound. The causes of subfertility and duration of subfertility were independent predictors for pregnancy during TI with ultrasound. Age and BMI were also independent predictive factors for pregnancy in subfertile couples with ovulatory factors. Both the CPRs and the CLBRs substantially increased during the first three cycles and 80% of pregnant patients conceived. The CPRs significantly increased until the fifth cycle but did not after the sixth cycle. The CLBRs significantly increased until the fourth cycle but did not after the fifth cycle. To our best knowledge, few studies have reported on the pregnancy outcomes of TI with ultrasound (31).

Many couples who have visited the clinic for subfertility care believe that they are already having targeted sexual intercourse through multiple information sources. However, only 13% of them correctly identified the fertile window (32). A study using data from the anonymous information stored in the cloud database from users of the Clearblue^®^ Connected Ovulation Test System (SPD Swiss Precision Diagnostics, GmbH, Geneva, Switzerland) showed that ovulation days could vary widely, even if the cycle length can be predicted with this system (33). In addition, it has been suggested that effective fertile windows are narrower in many subfertile couples (34, 35). Therefore, it is important to provide the exact fertility window to subfertile couples.

TI with ultrasound is a direct method to precisely predict the fertile window. However, there may be several disadvantages. Although the risk of subjective interpretations of the ultrasonic morphology of the ovaries is possible (29), bias by the physician might be minimal because our study was performed by an experienced infertility specialist. Ultrasound is relatively expensive and less accessible. However, these additional costs and efforts are counterbalanced by the potential to prevent overtreatment such as ART in couples who could conceive with TI (36). In addition, the cost of ultrasound is cheaper than that in other countries and is partially covered by national health insurance in Korea. TI with ultrasound has been widely used for subfertile couples with patent fallopian tubes and adequate semen parameters in Korea.

TI with ovulation induction is an effective treatment for women with ovulatory factors (36, 37). Women with higher AMH and/or irregular menstrual cycles had a better chance of pregnancy, and these characteristics were frequently shown in women with ovulatory factors. In the present study, the CPR was the highest at 39.8% and the CLBR was also higher with borderline statistical significance at 28.2% in subfertile couples with ovulatory factors when compared to subfertile couples with other causes. Old age and longer duration of subfertility were unfavorable independent predictors for pregnancy and higher BMI was a favorable independent predictor.

Although it is not clear that women with endometriosis may benefit from ovulation induction, it has been empirically used for subfertile women without absolute causes of infertility. There are several treatment options available to women with endometriosis who are seeking fertility. While not routinely recommended, expectant management is an option for women who are hesitant to pursue ovarian stimulation with or without IUI or *in vitro* fertilization (IVF). The fecundity rate of women with endometriosis is lower than non-infertile reproductive population. However, women with endometriosis can conceive without intervention. After surgical treatment, the choice between expectant management, empirical treatment, and IVF should be based on age, surgical results, and the severity of any other coexisting infertility factors. Treatment options for asymptomatic women with known or suspected minimal or mild endometriosis and no other infertility factors include expectant management, surgical treatment, empirical treatment, clomiphene or exogenous gonadotropins and IUI, and IVF (38, 39). If, therefore, women with endometriosis are eligible for timed coitus with ultrasound, they were included in the present study. In the present study, 30 of 36 patients with endometriosis were included after surgical treatment. Although previous studies suggested that surgery for endometriosis improved the chance of natural conception (40, 41), women with endometriosis had a significantly lower pregnancy rate than women with ovulatory factors. The CPR at 10months was significantly the lowest at 16.7% and the CLBR was also lower with borderline statistical significance at 13.9%. Our findings were in line with the results from the previous study (42). Few studies have compared the effects of TI according to infertility causes, but a study that assessed the pregnancy rate of those on the IVF waiting list showed interesting results (43). Women with endometriosis had the lowest hazard ratio to have a treatment-free ongoing pregnancy, indicating they will have the least effectiveness using non-ART strategies. The women with endometriosis should consider advancing earlier to the next treatment such as ART. Uterine factors including a uterine myoma larger than 6 cm or with distortion of the uterine cavity, adenomyosis, and endometrial polyp have been implicated in infertility. However, the clinical evidence and benefit of different management options for subfertility are also conflicting (44–46). TI with ovulation induction has been empirically used for subfertile women with normal ovulatory function including unexplained infertility, although it is not recommended as it is no more effective than expectant management (47). Our findings of CPRs and CLBRs may provide data on subfertility management for subfertile couples with nonovulatory factors in real–world setting.

The duration of subfertility has been used as a major factor to start infertility treatment. Several studies showed that the pregnancy rate was particularly compromised by the duration of subfertility in TI (31, 42, 48–50), a finding that agrees with our findings. Our study was similar to a prospective cohort study of couples with at least 1 year of unsuccessful attempts to conceive who undergo TI after FABM training. A duration of non-conception for more than 2 years significantly reduces the chances of pregnancy (p=0.006; OR: 0.38; 95% CI: 0.19-0.97) (42). In a randomized controlled trial using 2 cycles of TI and fertility awareness device measurement of the E1G and LH levels, more women who had been trying to conceive for <6 months became pregnant than those who tried to conceive for >6 months (OR: 2.67) (48). In the present study, couples with subfertility duration ≥24 months had a poor prognosis and the longer duration of subfertility was found to be an independent unfavorable factor in both couples with ovulatory factor and non-ovulatory factors. However, no significant difference was found in the pregnancy rates between the groups with a duration <12 months and those with a duration between 12 and 24 months. The cause may be due to the difference in the study participants. The present study included couples with subfertility duration >6 months, but the previous study included volunteers with favorable outcomes. Therefore, our findings suggest that TI with ultrasound for couples with a subfertility duration of less than 2 years may still be considered if they have no absolute indication for ART.

It is well recognized that fertility declines as age increases. In the present study, however, female age >35 years was not an independent factor to predict pregnancy in the multivariate logistic regression model. Logistic regression analysis for predicting pregnancy shows all age categories (37, 38, 39 and 40 years of age as a cutoff age) were not independent unfavorable predictors for clinical pregnancy in overall participants. Participants were subdivided into subfertile couples with ovulatory factor and non-ovulatory factors and logistic regression analyses were performed in each group. Age was the independent unfavorable predictor for clinical pregnancy in subfertile couples with ovulatory factor but still not in subfertile couples with non-ovulatory factors. Since, although it was clearly explained, the present study included subfertile couples with heterogeneous etiologies including uterine factor, endometriosis, and mild male factor, this finding may be attributed to the selection bias.

Cycle characteristics have been implicated in the success of ART (51, 52). However, cycle characteristics, including endometrial pattern and thickness, the presence of endometrial polyps, the number of follicles >14 or 18 mm, and whether hCG was administered did not affect pregnancy in the present study. Additionally, ovulation methods did not affect pregnancy.

ART is often regarded as a panacea and first-line treatment for those with difficulty in achieving pregnancy as they developed dramatically. There is no evidence-based guideline on the reasonable and sustainable duration of TI. Reliable estimates of cumulative probabilities are important in identifying appropriate thresholds to be used as indicators to advance to the next level of treatment.

Most studies of timed intercourse focused on ovulation prediction methods were conducted on women without known infertility, (53) or with a short period of trying to conceive (48). The effect of timed intercourse in fertile couples and subfertile couples is assumed to be different. The previous study showed that the quality of FABM like the vaginal discharge correlates well with the cycle-specific probability of pregnancy in normally fertile couples but less in subfertile couples (54). Approximately 85-90% of healthy young couples conceive within 1year, most within 6 months (1, 2). The present study included couples with sufficient duration for more than 6 months. Therefore, participants in the present study may have decreased reproductive efficiency. Timed intercourse using various ovulation prediction methods may be a reasonable recommendation for those couples. Although timed intercourse has been widely used for those couples in the daily fertility practice, data on predictive factors and cumulative pregnancy or live birth rate were still lacking, especially in timed intercourse using ultrasound. Our findings may provide the effectiveness on timed intercourse in subfertile couples without absolute infertile causes, such as bilateral tubal obstruction and premature menopause.

In the present study, the estimated CPRs at one, three, six, nine, and 12 cycle(s) were 9.8%, 22.5%, 26.7%, 27.0%, and 28.1%, respectively. Studies have estimated the CPR with TI using various methods under different settings. In a randomized controlled trial using urinary LH and the E1G kit, the CPR for two cycles was 22.7% (48). This study has limitations in that only 2 cycles were conducted and participants were excluded if they had been trying to conceive for > 2 years; the median time trying to conceive was short, only 8 months. Koo et al. (31) compared the likelihood of achieving pregnancy using TI with ultrasound according to the serum AMH levels. The clinical pregnancy rate of 202 women was 40.6% with up to 29 months of follow-up. The CLBRs differed according to the serum AMH level compared with 4.0%-26.0% at 6 months, 4.0%-37.7% at 12 months, and 17.2%-41.8% at 18 months. No results were obtained for the CPR at each cycle and for the CLBR of all the participants, limiting the ability to compare their findings with ours. However, a relatively high CPR and CLBR may be due to the favorable characteristics of the participants because women with other causes of subfertility, such as uterine factors, endometriosis, irregular menstruation, and polycystic ovary syndrome (PCOS), were excluded.

The Irish retrospective cohort study of 1,072 women undergoing TI with FABM found similar results to ours, with a CPR at 1 year of 25.9% (55). However, the CPR at 6 months was lower than ours at 14.1%, and long-term follow-up of up to 24 months presented a CPR of 33%. The study provided additional treatments, such as those to support luteal hormonal production and medication to enhance cervical mucus production. By comparison, a German prospective cohort study with 340 women undergoing TI with FABM demonstrated higher estimated CPRs of 38%, 68%, 81%, and 92% at one, three, six, and 12 cycles, respectively (2). The participants switched immediately from contraception to reproduction using TI; therefore, the first cycle using TI was also their first attempt for pregnancy. However, the average duration of time spent previously trying to pregnancy for the Irish study population was 5.6 years, which is greater than that of our study. These characteristics may be a major factor associated with the difference in CPR in each study. In the earlier studies, as the number of TI cycles increased, the rate of increase in the CPR tended to decrease. In a study conducted on TI based on the method of calendar calculations, the maximal pregnancy rate was approximately 30% per cycle in the first two cycles and then progressively decreased (56). In an observational study, the estimated CPR was analyzed for 340 women who had undergone TI with FABM. Most pregnancies occurred within six cycles (2), and other studies also suggest at least six cycles before any intervention (57, 58). In the present study, the CPR and the CLBR substantially increased during the first three cycles and 80% of pregnant patients conceived. The CPR and the CLBR also significantly increased until the fifth and fourth cycle, respectively, which may be considered as valid findings that was not statistically disclosed in other studies. Our findings suggest that three cycles of TI are highly recommended and that performing only up to six cycles is likely appropriate because the pregnancy rate does not increase significantly after the sixth cycle.

The present study has several limitations. First, more than half of the patients (58.2%; 166/285) withdrew from treatment before six cycles of treatment. After the 9th cycle, the number of patients was very small in each cycle. Because TI is a conservative or initiating treatment in practice, dropout is difficult to avoid in long-term follow-up studies like ours due to the tendency to advance to the next-step treatment. Many patients were dropped out for various reasons including pregnancy, starting advanced infertility treatment, and loss of follow-up. Several previous studies did not report the dropout rate, while others have reported 55% for up to five cycles (59) and 64% (60) for up to six cycles in cohort studies of ART. Therefore, the dropout rate from our study is comparable to that from previous studies. Second, heterogeneity in subfertile causes could be a major limitation of the present study. Since, however, many infertile couples suffer from multiple etiologies, data on subfertile couples with homogenous cause may not provide sufficient information to those couples. Therefore, the present study may provide the information on the effectiveness of timed intercourse of subfertile couples in real-world setting. Third, the present study had not control or comparator group. Since the aim of this study was to determine predictive factors for pregnancy and assess the CPR in subfertile couples undergoing TI with ultrasound, the outcomes between TI using ultrasound monitoring were not compared with those using other methods or no treatment. In the previous study, cycle fecundability was 4.9%(55 pregnancies/1123 cycles) when subfertile couples conducted the fertility awareness training through tracking basal body temperature and cervical secretions (42). Also untreated patients with unexplained infertility have a cycle fecundability ranging typically between 2% and 4% (61). In fact, our study could have different patient characteristics from those of previous studies, which makes it difficult to directly compare cycle fecundability with those of previous studies. Considering, however, that the cycle fecundability was 9.4% in our study, TI with ultrasound monitoring may be a valuable option for subfertile patients. Fourth, insufficient power was the limitation of the present study. A power calculation was performed using PAWE (Power for Association with Error) software version 1. 2. (http://linkage.rockefeller.edu/pawe/pawe.cgi). A total of 406 patients (114 in pregnant group and 292 in non-pregnant group) would be needed to detect a 6-month difference in the duration of infertility between pregnant and non-pregnant group with a significance level of 0.05 and 80% power when the pregnancy rate was assumed to be 25%. Our sample size has the power of 66% to detect a significant difference for the duration of infertility. To our best knowledge, however, few studies have reported on the pregnancy outcomes of TI with ultrasound. Moreover, there has been no randomized trial in this issue. In spite of several weaknesses of the present study, therefore, our study might provide meaningful findings in a real-world setting for subfertile patients eligible for timed coitus. In the future, additional randomized controlled trials on a larger scale would be necessary to confirm our findings.

In conclusion, we found that the duration of non-pregnancy for more than 2 years and endometriosis reduced the chances of pregnancy during TI with ultrasound. In subfertile women with ovulatory factors, old age ≥35 years may be an unfavorable predictor of pregnancy and higher BMI ≥ 25 kg/m^2^ may be a favorable predictor during TI with ovulation induction using ultrasound monitoring. TI with ultrasound of three cycles was an acceptable option for subfertile couples without absolute indication of ART. However, couples who failed to achieve conception by the fourth or fifth cycle of TI with ultrasound may be encouraged to consider advancing to the next treatment strategy. This study provided useful information on predictive factors of conception, CPR and CLBR in subferile couples undergoing TI with ultrasound. Physicians should provide individualized approaches to patients with further refinement based on the number of treatments to avoid over- and under- treatment. In future work, large prospective control studies are needed to confirm these findings.

## Data Availability Statement

The original contributions presented in the study are included in the article/supplementary material. Further inquiries can be directed to the corresponding author.

## Ethics Statement

The studies involving human participants were reviewed and approved by Institutional Review Board of Severance Hospital, Yonsei University College of Medicine. Written informed consent for participation was not required for this study in accordance with the national legislation and the institutional requirements.

## Author Contributions

Conceptualization, YC and SA. Methodology, YC, SC and SA. Software, JP and IL. Validation, SS and HK. Formal analysis, YC and SA. Investigation, SA, YP, HB and HK. Resources, YC, BY, JL and IL. Data curation, YC and SA. Writing—original draft preparation, SA. Writing—review and editing, YC and SA. Visualization, YC and SA. Supervision, YC, SC. Project administration, YC, SC and BL. Funding acquisition, YC and SC. All authors contributed to the article and approved the submitted version.

## Funding

This study was supported by the Korea Health Technology R&D Project through the Korea Health Industry Development Institute, funded by the Ministry of Health & Welfare, Republic of Korea (HI18C2047).

## Conflict of Interest

The authors declare that the research was conducted in the absence of any commercial or financial relationships that could be construed as a potential conflict of interest.
